# Study of Δ9-tetrahydrocannabinol (THC) and cannabidiol (CBD) extraction FROM dried oral fluid spots (DOFS) and LC–MS/MS detection

**DOI:** 10.1186/s42238-021-00088-8

**Published:** 2021-07-12

**Authors:** Roberta Gorziza, Joseph Cox, Renata Pereira Limberger, Luis E. Arroyo-Mora

**Affiliations:** 1 Department of Pharmacy, Federal University of Rio Grande Do Sul, Av. Ipiranga 2752, Porto Alegre, RS 90610-000 Brazil; 2 Department of Forensic and Investigative Science, West Virginia University, 302 Oglebay Hall, Morgantown, WV 26506 USA

**Keywords:** Dried oral fluid spots, DOFS, Drug detection, THC, CBD, LC–MS/MS

## Abstract

**Background:**

Oral fluid is a widely studied matrix able to isolate the primary Cannabis constituent THC, facilitating its detection via mass spectrometry, and in most cases link these findings to recent drug use. As an alternative to liquid oral fluid, dried oral fluid spots (DOFS) is a simple and a low-cost sampling technique. It has shown improved stability compared to liquid samples, allowing for the possibility to preserve the specimens under various temperature and humidity conditions. The sampling strategy is straightforward and involves the application of a small quantity of oral fluid aliquot to a paper substrate that is set to air dry allowing for on-site collection at a large-scale demand. The goal of this study is to study THC and CBD extraction from DOFS, applying a previous established protocol for a LC–MS/MS qualitative method validation. Although other drugs of abuse have been included in DOFS methods, this is the first method validation including cannabinoids. An alternative oral fluid extraction method (WAX-S tips) is demonstrated to improve the recovery of the analytes.

**Methods:**

A pool of blank oral fluid was used to prepare THC and CBD spiked DOFS samples for method validation and application. Spiked oral fluid was used to demonstrate WAX-S tips THC and CBD extraction. All samples were analyzed on a LC–MS/MS instrument.

**Results:**

The qualitative method validation for THC and CBD confirmation in DOFS included method selectivity, matrix effects (< 20%), recovery (average of 25%), process efficiency (average of 21%), LOD (2 ng/mL for THC and 4 ng/mL for CBD), absence of carryover, and DOFS stability (70% in 35 days) as figures of merit. The method application in blindly prepared samples demonstrated the method capability to identify THC and CBD. WAX-S tips extraction showed an average of 91% recovery of THC and CBD from liquid oral fluid.

**Conclusions:**

THC and CBD extraction from DOFS showed low recoveries. However, the LC–MS/MS qualitative confirmation of THC and CBD in DOFS could improve cannabinoids screening in oral fluid, as it shows adequate LOD and stability over time. This method has potential for assisting the screening of drivers under possible drug influence by facilitating sample transportation and temporary storage in dried spot form. Additional research is suggested for WAX-S tips extraction and quantitative method validation.

## Background

Δ9-tetrahydrocannabinol (THC) is the main product of cannabis, known for its psychoactive effects, and cannabidiol (CBD) is the second major component of the plant (Russo and Guy 2006). CBD, combined with low THC concentrations, is associated with therapeutic effects (Russo and Guy 2006; Pisanti et al. 2017). Regulations for cannabis medical products vary among countries and states (Abuhasira et al. 2018) and there is a growing concern regarding the control of cannabis legal products, CBD based, which may contain higher THC levels than permitted (White 2019). Although the relation between cannabis medical use and driving safety is still limited, there are indications that its use has increased the prevalence of driving under the influence of cannabis (DUIC) (Fink et al. 2020).

Oral fluid is accepted as an adequate matrix for drug detection, offering valuable correlations with drug concentrations in blood (Cone and Huestis 2007). Oral fluid collection is not invasive as blood collection, and it can be performed by non-medical personnel, like police officers, therefore facilitating on-site collection (Drummer 2008; Walsh et al. 2008). In addition, the oral mucosa is exposed to high THC concentrations during smoking, the principal route of cannabis administration (Huestis 2007). For this reason, THC is the substance of choice to detect cannabis use in oral fluid. The European guideline Driving Under Influence of Drugs—DRUID (Schulze et al. 2012) recommended the value of 27 ng/mL as cut-off for THC detection in oral fluid, while the North American agency Substance Abuse and Mental Health Services Administration (SAMHSA 2019) established lower cut-offs values (4 ng/mL for screening tests and 2 ng/mL for confirmatory tests) for THC detection in oral fluid. Lower cut-offs are necessary considering that THC concentrations in oral fluid decrease fast with time, after smoking. A study, performed by Huestis and Cone (Huestis and Cone 2004), detected 5800 ng/mL of THC in oral fluid after 0.2 h of smoking; then, after 0.33 h, the concentration decreased to 81 ng/mL, reducing to less than 0.1 ng/mL after 12 h. Similarly, Milman et al. (2012), detected 22,370 ng/mL of THC after 0.25 h of smoking, and after 6 h the concentrations decreased significantly (0.9–90.4 ng/mL), reducing to lower concentrations in 22 h (0.4–10.3 ng/mL). Thus, THC identification in oral fluid is indicative of recent drug use due to its short detection times (Huestis 2007). The demonstration of recent drug use in oral fluid is valuable in particular situations, such as the screening for potential drivers under drug influence, the workplace testing, and anti-doping programs (Lee and Huestis 2014). This is not only to prevent accidents and prohibited use but also for post-accident or post-event evaluation of those involved in accidents (Lee and Huestis 2014).

Dried Matrix Spots (DMS) have been of interest in forensic toxicology (Chepyala et al. 2017; Sadler Simões et al. 2018; Caramelo et al. 2019; Ribeiro et al. 2019; Seymour et al. 2019; Gorziza et al. 2020), especially to simplify on-site sample collection while reducing time and resources. It consists of applying a small quantity of a biological sample (e.g., 50 µL) into a paper substrate, and set it to dry (Hannon and Therrell 2014; Resano et al. 2018). The simple approach facilitates sampling from collection sites located offsite from the actual laboratory where the analytical work is performed. This is particularly useful for DUIC screening. DMS are also designed for a simple and fast sample extraction, reducing resources cost and chemical waste (Déglon et al. 2015). When coupled to sensitive detection instruments, such as liquid chromatography tandem mass spectrometry (LC–MS/MS), it can provide drug detection at lower concentrations (Gorziza et al. 2020). THC identification and quantification have been shown in analytical methods using dried blood spots (DBS) (Thomas et al. 2012; Mercolini et al. 2013; Kyriakou et al. 2016; Protti et al. 2017), but THC isolation have been studied only for an extraction protocol using dried oral fluid spots (DOFS) (Stoykova et al. 2016).

Therefore, the aim of this study is to include THC and CBD detection to an established DOFS sampling protocol and an extraction procedure (Gorziza et al. 2020), which covered the identification and quantification of amphetamine, methamphetamine, benzoylecgonine, ketamine and mitragynine, using a LC–MS/MS instrument. To the best of our knowledge, CBD has been included for the first time in DMS, and it can help to evaluate cannabis medical use, among other scenarios. Additionally, disposable tips containing Weak Anion Exchange and Salt (WAX-S tips) were evaluated for the extraction of THC and CBD from oral fluid.

## Methods

### Chemicals and materials

THC, CBD, THC-d3 and CBD-d3 standards were acquired from Cerilliant (Round Rock, TX, USA). Methanol and acetonitrile—Optima® LC/MS Grade, ammonium formate and formic acid were purchased from Fisher Scientific (Waltham, MA, USA). Ultrapure water was obtained using a Direct-Q 3UV system of Millipore (Burlington, MA, USA). Whatman 903® paper was acquired from GE Healthcare Life Sciences (Marlborough, MA, USA) and WAX-S tips (300µL Hamilton 2 mg WAX + 10 mg salt) were purchased from DPX technologies (Columbia, SC, USA).

### Instrumentation

The instrument for data acquisition was an Agilent Technologies Liquid Chromatography 1290 Infinity II coupled to an Agilent 6470 triple quadrupole MS/MS (Agilent Technologies, Santa Clara, CA), operated in positive electrospray ionization mode, ESI ( +).

Chromatographic separation of THC and CBD was obtained with a Zorbax RRHD C18 column (3.0 × 50 mm, 1.8 μm) from Agilent (Santa Clara, CA, USA), using a gradient elution of 0.1% formic acid and 5 mM ammonium formate in water (solvent A), and acetonitrile with 0.1% formic acid (solvent B). The gradient was set with an initial flow of 95% solvent A for 0.5 min, reduced to 70% at 2 min and to 65% over 3 min; then, at 4 min, it was reduced to 50%, and at 7 min, it was reduced to 5%, in a total run of 10 min. The volume of injection was 1 µL.

Table 1 shows THC and CBD transitions established for the dynamic multiple reaction monitoring (dMRM) method, as well as their retention times. The monitored transitions were chosen using Agilent MassHunter Optimization software.

**Table 1:** Monitored transitions (*m/z*) for Δ9-tetrahydrocannabinol (THC) and cannabidiol (CBD) standards in methanol, optimized using the Agilent MassHunter Optimization software for liquid chromatography tandem mass spectrometry (LC–MS/MS). Retention times were established in the chromatography separation

Drug	Associated Internal Standard (ISTD)	*m/z* Q1	*m/z* Q3	Retention Time (Minutes)
CBD	CBD-d3	315.2	315.2 → 123 315.2 → 193	8.22
THC	THC-d3	315.2	315.2 → 123 315.2 → 193	9.13

### DOFS preparation and extraction procedures

DOFS sample preparation and extraction procedures were conducted as previously described (Jacques et al. 2019; Gorziza et al. 2020).

Blank oral fluid was obtained from laboratory staff volunteers. It was requested for them to not consume food and/or drinks at least one hour prior to the collection, which was performed by direct spitting into non-identified polypropylene tubes. No personal information was requested, and all samples were pooled into a single container. The pooled oral fluid was kept at 6 ºC for no longer than a week.

Previously cut pieces of Whatman 903® filter paper (1,6 cm × 1,6 cm) were placed on a surface covered in aluminum foil, previously identified for each sample. Spots were spiked with 50 µL of blank oral fluid and it was allowed to dry for at least 2.5 h at room temperature. Afterwards, the spots containing dried oral fluid were spiked with 50 µL of a drug mix with THC and CBD, daily prepared at specific concentrations for validation procedures, and it were allowed to dry for at least 1.5 h at room temperature.

DOFS extraction was proceeded as it follows: a) the filter paper (1.6 cm × 1.6 cm) was folded and transferred to a polypropylene tube; b) 1 mL of extracting solvent (methanol: acetonitrile 50/50) was added; c) samples were submitted to 10 min of sonication; d) samples were submitted to 10 min of centrifugation, at 10,000 rpm; d) the supernatant was transferred to a glass vial and dried with a gentle nitrogen stream at 37 ºC to prevent over drying; e) 100 µL of reconstitution solution (95 µL of methanol and 5 µL of an internal standard mix solution at the concentration of 1 µg/mL (final concentration of 50 ng/mL) was added; f) samples were subjected to a vortex for 10 s; g) 1 µL of sample was injected in the LC–MS/MS for analysis.

### WAX-S tips extraction

Into a 1.5 mL polypropylene tube, 50 µL of oral fluid spiked with THC and CBD (at the concentration of 12 ng/mL), 100 µL of acetonitrile and 5 µL of an internal standard solution (1 µg/mL solution of THC-d3 and CBD-d3, with a final concentration of 50 ng/mL) were added. Using a 300 µL WAX-S tip and a micropipette, the mix was aspirated and dispensed for three times. Then, 100 µL of the top layer, a hydrophobic phase containing the analytes, was transferred to a glass vial and 1 µL of sample was injected in the LC–MS/MS for analysis.

### Qualitative confirmation method validation

The Standard Practices for Method Validation in Forensic Toxicology guideline (American Academy of Forensic Science (AAFS) Academy Standard Board (ASB). Standard Practices for Method Validation in Forensic Toxicology (2019)) established the required parameters for qualitative confirmation/identification methods: carryover, interference studies, ionization suppression/enhancement, limit of detection and processed sample stability if applicable. Following these requirements, our qualitative method validation included carryover, selectivity and interference studies, ionization suppression/enhancement (matrix effects) limit of detection (LOD), and stability as figures of merit. Additionally, extraction recovery and process efficiency were also evaluated.

Selectivity and interferences were evaluated using three different approaches. Initially, THC, CDB and their respective internal standards were injected individually. Considering that the optimized transitions for CBD and for THC were the same, these compounds were differentiated by their retention times in chromatography (8.22 and 9.13 min, respectively). The second approach evaluated a pool of blank oral fluid from ten different volunteers, in triplicates, with and without internal standards. THC and CBD’s absence were then checked on these samples. Finally, common compounds (Table 2) were subjected to the method to check for possible interferences.

**Table 2:** List of compounds (methanolic standards) evaluated for possible interferences in the liquid chromatography tandem mass spectrometry (LC–MS/MS) method for Δ9-tetrahydrocannabinol (THC) and cannabidiol (CBD) identification

Class	Compounds
**Opioids**	6-Acetylmorphine
	Oxycodone
	Hydrocodone
	Buprenorphine
	Norbuprenorphine
	Ethylmorphine
**Synthetic cannabinoids**	JWH-018
	JWH-073
	XLR-11
	AB-FUBINACA
	AB-PINACA
	MAM2201
**Stimulants**	Amphetamine
	Methamphetamine
	Cocaine
**Dissociative Anesthetic** **Other alkaloids** **Supplements**	Ketamine
	Mitragynine
	1S,2R ( +)- Ephedrine
	Methylphenidate
	Sibutramine
	Caffeine
	Synephrine
	Octopamine
	Methylhexanamine (DMAA)

Matrix effects, extraction recovery and process efficiency were evaluated as previously described by Matuszewski et al. (2003). For these experiments, DOFS samples were prepared as described in section DOFS Preparation and Extraction Procedures, using a pool of blank oral fluid from seven different volunteers. Three sets of samples were prepared at administratively determined low and high concentrations (12 ng/mL and 50 ng/mL, respectively): a) 6 replicates of neat standard solutions in methanol; b) 10 replicates of DOFS samples fortified after extraction; c) 10 replicates of pre-spiked DOFS samples. Afterwards, the mean peak areas for each set were used to calculate matrix effects (ME), process efficiency (PE) and recovery (RE) percentages, according to the formulas:

$$\mathrm{ME}: \left(\frac{b}{a} \right)\mathrm{x }100\mathrm{ PE}: \left(\frac{c}{a}\right) \mathrm{x }100\mathrm{ RE}: \left(\frac{c}{b}\right)\hspace{0.17em}\times \hspace{0.17em}100$$

WAX-S tips were also studied for THC and CBD extraction from oral fluid. For these experiments, samples were prepared using a pool of blank oral fluid obtained from seven different volunteers. In this matter, ME, PE and RE were calculated, as previously described above, using a) 6 neat standards solutions in methanol, b) 10 post-spiked samples, and c) 10 pre-spiked samples. As a demonstration, WAX-S tips were evaluated only for a low concentration (12 ng/mL).

The LOD was determined by fortifying DOFS samples (prepared with a pool of blank oral fluid from seven different volunteers), at decreasing concentrations (4, 2, 1 and 0.2 ng/mL) for at least three runs. LOD was defined as the lowest concentration at which the signal-to-noise (S/N) ratio was equal or greater than 3.3, and it could be also visually determined from chromatographic peaks analysis and THC and CBD monitored transitions (Table 1).

Carryover was assessed by injecting three blank matrix samples (prepared with a pool of blank oral fluid from seven different volunteers), after extracted DOFS samples containing 100 ng/mL of THC and of CBD. It was considered insignificant if the LOD criteria were not met.

Finally, a triplicate of DOFS samples (prepared with a pool of blank oral fluid from seven different volunteers) was prepared, spiked at a medium concentration of 30 ng/mL, and dried overnight, therefore kept in a plastic bag at 6 ºC. After 35 days, these samples were extracted as previously described in section DOFS Preparation and Extraction Procedures, and compared to freshly prepared and extracted DOFS samples, to evaluate DOFS stability.

### Method application

As authentic samples were not available to evaluate the validated method, a blind study with simulated DOFS samples (n = 8) was conducted. A pool of blank oral fluid (n = 7) was applied on the filter papers (50 µL), it was allowed to dry (2.5 h) and therefore it was spiked with different THC and/or CBD concentrations, higher than their LODs (2 and 4 ng/mL, respectively). A researcher, different from the one that proceeded with the sample extractions and analysis, prepared eight different drug mixes to spike the DOFS samples (50 µL). After drying (1.5 h), the spiked DOFS samples were extracted as previously described. After sample extraction and analysis, the eight samples were evaluated by the researcher as “positive” or “negative” for THC and for CBD, and further compared to the blindly prepared drug mixes.

## Results

### Chromatography

**Fig. 1:**
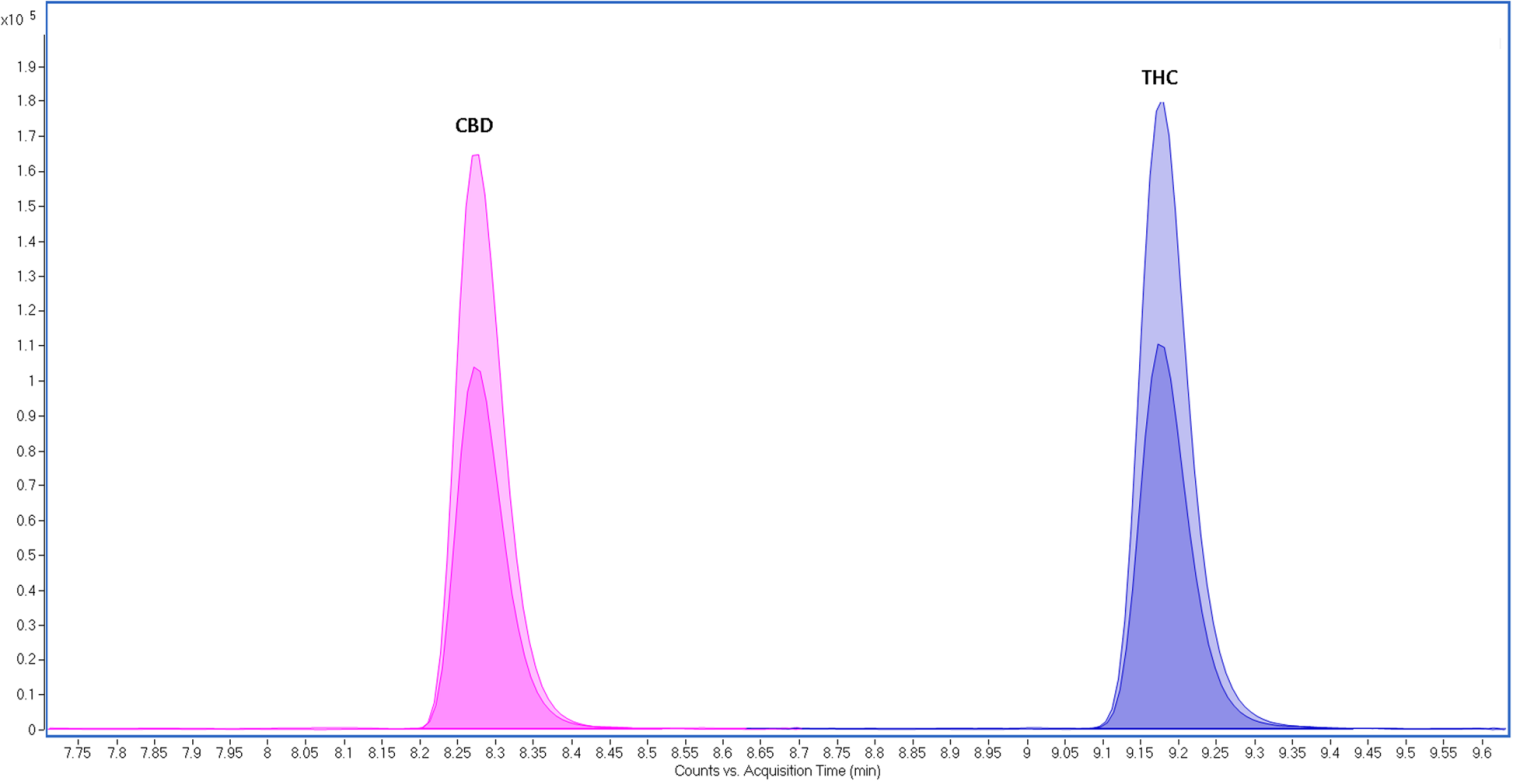
CBD and THC chromatographic separation at 8.22 and 9.13 min, respectively. The chromatogram was extracted from an injection of a methanolic solution at 25 ng/mL

Initially, the chromatographic separation for the isolation of THC and CBD was evaluated. The method has presented proper selectivity for THC and CBD. Both analytes were identified by the same transitions (Table 1), therefore their elution should occur at different retention times in chromatography. Figure 1 shows CBD eluted at 8.22 min and THC at 9.13 min. The chromatographic separation allowed for adequate CBD and THC visual discrimination.

### Sample preparation

The first step for DOFS sample preparation is to define the adequate filter paper, its size, its capability of oral fluid absorption and the amount of time needed for complete dryness. Jacques et al. (2019) studied these conditions for Whatman 903® filter paper, defining that a 1.6 cm × 1.6 cm piece of paper would be necessary to absorb 50µL of oral fluid. This sample require a minimum of 2.5 h to dry. Our protocol utilized this study as a reference for these sample preparation parameters.

The second aspect of DOFS sample preparation is oral fluid collection. To simplify and to reduce the costs for this procedure, the collection was performed by direct spitting of neat oral fluid into polypropylene tubes. Neat oral fluid is a viscous sample, and it is an intrinsic factor in authentic samples. However, it is an aspect of concern in method validation. Gorziza et al. (2020) discuss how previous DOFS studies handle drug spiking in oral fluid, reporting the use of artificial, frozen or centrifugated oral fluid. These procedures do not represent the practical scenario for sample collection. Therefore, based on Numako et al. (2016) study, Gorziza et al. (2020) propose a two-step DOFS sample preparation, by adding total oral fluid on the filter paper and, after drying, adding the methanolic mix of drugs. The two-step process is necessary to respect the filter paper absorption capability of only 50 µL at a time. This procedure utilizes authentic oral fluid, and it guarantees the drug quantities for method validation, avoiding dilution in viscous samples. Thus, our sample preparation followed Gorziza et al. (2020) two-step protocol.

DOFS sample extraction was also performed as previously described for other five drugs of abuse (Gorziza et al., 2020). However, extraction using methanol as solvent and glass materials were also tested, following previous studies with DBS (Mercolini et al. 2013; Kyriakou et al. 2016; Protti et al. 2017). These modifications did not perform better than the initial protocol.

WAX-S tips are disposable tips containing weak anion exchange (WAX) sorbent and salt (S), allowing for a salting-out assisted liquid–liquid extraction (SALLE). Although WAX tips are recommended for strong and weak acids analysis (DPX Technologies), when combined with salt for SALLE, WAX-S tips are recommended for hydrophobic compounds, such as cannabinoids (DPX Technologies). The sample preparation includes the pre-treatment with acetonitrile, for protein precipitation. It was added 100µL of acetonitrile to 50µL of spiked oral fluid. The extraction involves the steps of conditioning, mixing the sample with the loose sorbent inside the tip, followed by activation, with the analytes binding to the sorbent, and the washing of matrix interferences. Finally, the analytes of interest are eluted in acetonitrile (DPX Technologies). The extraction and the elution steps occur by aspirating/dispensing all 150µL of liquid for three times. This method has been successfully applied for cannabinoids extraction from blood (Scheidweiler et al. 2016) and from urine (Andersson et al. 2016); the reported cannabinoid recoveries are between 54–84.4% and 42.4–81.5%, respectively. Therefore, it demonstrated satisfactory results in our study with oral fluid.

### Qualitative confirmation method

The tests for selectivity showed that the LC–MS/MS method was capable to properly identify THC and CBD. No interferences from endogenous peaks or signal contribution from internal standards were observed. In addition, different compounds with potential to be found in oral fluid (opioids, synthetic cannabinoids, stimulants, supplements, a dissociative anesthetic and an alkaloid – Table 2) were subjected to the method, and no interfering peaks for THC and CBD were observed.

After the establishment for the detection method, matrix effects, extraction recovery, process efficiency, LOD, carryover, and DOFS stability were evaluated as figures of merit for the qualitative method validation.

Matrix effects, recovery and process efficiency were studied for DOFS samples and subsequently for the WAX-S tips extraction method. All the results are shown in Table 3. Ion suppression was observed for THC and for CBD using both methods (DOFS and WAX-S tips), but these values are lower than 25%. The analyte’s average recovery was 25% and 91% for DOFS and WAX-S tips methods, respectively, along with an average process efficiency of 21% and 82% for DOFS and WAX-S tips methods, respectively.

**Table 3:** Matrix effects, absolute recovery and process efficiency for Δ9-tetrahydrocannabinol (THC) and cannabidiol (CBD), using dried oral fluid spots (DOFS) and weak anion exchange and salt (WAX-S) tips extraction

Drug	Matrix Effects* (%)	CV (%)	Absolute Recovery* (%)	CV	Process Efficiency * (%)	CV (%)
**THC (N = 10)**						
**DOFS**						
Low (12 ng/mL)	98.7	15.6	23.8	14.2	23.5	19.3
High (50 ng/mL)	80.3	17.3	25.3	13.9	20.3	20.7
**WAX-S tips**						
Low (12 ng/mL)	92.4	6.8	90.7	6.2	83.8	4.0
**CBD (N = 10)**						
**DOFS**						
Low (12 ng/mL)	75.2	18.5	24.8	18.0	18.6	23.9
High (50 ng/mL)	76.4	18.7	28.7	17.6	21.9	23.8
**WAX-S tips**						
Low (12 ng/mL)	87.5	7.0	93.2	8.7	81.6	5.5

^*^Matrix effects: accessed by dividing post-spiked average and neat samples, multiplied by 100;

^*^Process efficiency: accessed by dividing pre-spiked samples by neat samples, multiplied by 100;

^*^Recovery: calculated dividing pre-spiked samples by post-spiked samples, multiplied by 100

The next parameter evaluated for the DOFS method validation was the LOD, which was defined after analyzing six replicates at 0.2, 1, 2 and 4 ng/mL, for THC and for CBD. For THC the LOD was established at 2 ng/mL and, for CBD, it was established at 4 ng/mL. These concentrations showed signal-to-noise (S/N) values higher than 3.3, as well as their peak bases could be visually determined by the analyzer. After defining the LODs values, carryover was assessed by injecting blank oral fluid samples (n = 3) immediately after a high concentration of THC and of CBD (100 ng/mL). No carryover was observed, considering that the LOD criteria were not met by the blank oral fluid samples. Finally, DOFS stability was evaluated for a 35 days period. DOFS samples (at a concentration of 30 ng/mL) were kept at 6 °C and then extracted and compared to freshly prepared DOFS samples. Considering this period, a 30% loss was observed for THC and CBD.

### Method application

Once the method validation was complete, a blind study with DOFS spiked samples was conducted to evaluate its fits of purpose. Eight simulated samples were prepared and analyzed for THC and for CBD identification. The target analytes were correctly identified and confirmed in all samples: three samples were positive for THC; three different samples were positive for CBD and two samples were negative for both compounds.

## Discussion

A complete qualitative confirmation method is presented for THC and CBD identification in DOFS samples, coupled to a LC–MS/MS method. Additionally, a blind study with simulated samples was conducted as a proof of the method capability.

DOFS sampling and extraction has been successfully demonstrated in validated quantitative methods for different drugs of abuse and/or its metabolites detection: amphetamine, methamphetamine, 3,4-methylenedioxymethamphetamine (MDMA), cocaine, benzoylecgonine, cocaethylene, ketamine, mitragynine, methadone and 2-ethylidene-1,5-dimethyl-3,3-diphenylpyrrolidine (EDDP) (Jacques et al. 2019; Ribeiro et al. 2019; Gorziza et al. 2020). DOFS is an easy and practical sampling technique, however drug extraction from the filter paper is also an analytical challenge. Compound recovery will depend on a proper sample extraction protocol, as well as on each analyte affinity with the filter paper. For example, in a single DOFS extraction protocol the average recovery for amphetamine, methamphetamine, ketamine and benzoylecgonine was 82%, while the average recovery for mitragynine was only 55% (Gorziza et al. 2020). Similarly, Ribeiro et al. (2019) have obtained 67% of average recovery for methadone, and 54% for EDDP. Both mitragynine and methadone are lipophilic drugs (Gallagher 2009; Ramanathan et al. 2015), which could be one of the reasons for a higher interaction with the filter paper, leading to poor recoveries ratios (< 70%).

Cannabinoids are highly lipophilic drugs (Huestis 2007) and its adsorption onto plastic containers (Molnar et al. 2013) have been linked to poor cannabinoids recovery and stability in oral fluid, depending on the materials and/or buffers of sample collection devices (Lee and Huestis 2014). The extraction of THC and CBD from DOFS samples have shown limitations as well. After experimentally applying a successful extraction protocol of other drugs of abuse from DOFS (Gorziza et al. 2020) to include THC and CBD extraction, it was found a 25% average recovery for these compounds, along with a 21% average of process efficiency (Table 3). Both recovery and process efficiency are calculated from pre-spiked samples peak areas, divided by post-spiked samples and by neat samples peak areas, respectively (Matuszewski et al. 2003). While recovery considered the oral fluid effect, process efficiency compared DOFS samples extraction to accurate concentrations of drugs in methanol, so it is expected to observe similar, but lower values for process efficiency. The difference between these parameters represents the matrix effects, which showed an average value of 16% of ion suppression in DOFS (Table 3), and it is an acceptable value.

Stoykova et al. (2016) has studied THC extraction from DOFS for the first time. In this study, THC along with amphetamine, methamphetamine, 3,4-methylenedioxy-methamphetamine, cocaine, morphine, methadone, and clonazepam was spiked in oral fluid and therefore spotted onto a non-specified filter paper for drying. The extraction of these drugs from the filter paper using ethyl acetate and 1 M sodium hydroxide as an extraction solvent have shown > 70% recovery for all compounds, except for THC and clonazepam. For this reason, an extra DOFS pre-treatment (with methanol and 0.1 M hydrochloric acid) was added to achieve a 45% THC recovery, which is still a low percent value.

THC recovery from filter papers has also been studied using blood as a matrix (DBS). In 2012, Thomas et al. (2012) presented an extraction protocol from dried blood in a TNF Sartorius Card paper. This protocol utilizes a mixture of methanol and tert-butyl-methyl-ether solvents as a first extraction solution, followed by 45 min sonication and a final step of centrifugation for 5 min. Afterward, a second extraction with acetone was performed including another 30 min of sonication. Besides using this extensive extraction protocol, only a 19% recovery from the dried blood on filter paper was reported, a figure even lower than the one we have found in our DOFS protocol (25%). However, in the following years, three different studies reported recoveries > 80% THC when using DBS (Mercolini et al. 2013; Kyriakou et al. 2016; Chepyala et al. 2017). The extraction protocol followed by these research groups are described on Table 4. Attempting to improve THC and CBD recoveries obtained from our DOFS extraction protocol, we have evaluated these previously studied protocols, analyzing which filter paper types and extraction solutions they have utilized, as well as their extraction procedures details (e.g. tube material, vortexing, centrifugation, sonication, solvent evaporation).

**Table 4:** Comparative Δ9-tetrahydrocannabinol (THC) extraction protocols from dried blood spots, of previous studies

**Reference**	Thomas et al. (2012)	Mercolini et al. (2013)	Kyriakou et al. (2016)	Protti et al. (2017)
Paper Type	Sartorius TNF Card®	Whatman 903®	Whatman 903®	Whatman FTA™ DMPK-C Card®
Extraction Solution	Step 1. 100µL of MeOH^a^ + 400µL of TBME^b^ Step 2. 300µL of acetone	1 mL of MeOH^a^	990µL of MeOH^a^ and 10µL of IS^b^	1 mL of MeOH^a^
Tube	Polypropylene	Vial	Not informed	Vial
Procedures	Step 1. 45 min of sonication and 5 min of centrifugation (13,000 × g) Step 2. 30 min of sonication	1 min of vortexing and 5 min of centrifugation (1400 × g)	15 min of sonication and 5 min of centrifugation (3500 × g)	1 min of vortexing and 5 min of centrifugation (4000 rpm)
Solvent Evaporation	Vacuum centrifuge, 40ºC	Vacuum	Vacuum	Nitrogen Stream
Extraction Recovery	19%	83%	81%	92%

^a^MeOH: methanol; ^b^TBME: tert-butyl-methyl-ether; ^c^IS: internal standards

While working with dried spots it is essential to choose an appropriate filter paper to avoid inter-samples variation, and to optimize an extraction solvent to elute each target analyte from the paper, as well as time of extraction, and sonication (Zakaria et al. 2016). In the four previous studies with THC on DBS, three different types of filter paper were chosen, including Whatman 903®, the one utilized in our DOFS study. After guaranteeing the filter paper quality to spot samples and allowing them to dry, analytes recoveries depend on standardizing an adequate elution from the paper and on minimizing the compounds losses and/or degradation through the procedure (Zakaria et al. 2016). In regards to extraction efficiency, the studies that present higher THC percent recoveries (Mercolini et al. 2013; Kyriakou et al. 2016; Protti et al. 2017) reported the use of pure methanol as extraction solvent, also using a short period of time (5 min) of centrifugation for THC extraction (Table 4). Of these studies, only Kyriakou et al. (2016) added a sonication step (15 min) to their protocol which was also applied to our DOFS extraction protocol to improve the analyte elution. We have experimentally tested the use of pure methanol in our DOFS protocol, applying 10 min of sonication following 10 min of centrifugation, and it did not perform better than a mixture of methanol and acetonitrile (50/50, v/v), initially utilized in our protocol, for THC or for CBD extraction. The mixture was then chosen considering that THC and CBD detection could be added into a protocol for concomitant detection of a major pool of drugs of abuse, as previously described (Gorziza et al. 2020). As for inherent factors through the extraction procedure, previous studies have observed THC and CBD loss to plastic containers during the experiments (Molnar et al. 2013). In this matter, it was noticed that Mercolini et al. (2013) and Protti et al. (2017) describe their experiments using a glass vial (Table 4), and then we conducted an experiment for DOFS extraction avoiding all kinds of plastic, including tubes and pipette tips. However, THC and CBD recoveries remained the same (25% and 26%, respectively). A second hypothesis for THC and CBD loss would be their degradation over the process of solvent drying using a nitrogen stream. Nonetheless, while Mercolini et al. (2013) and Kyriakou et al. (2016) used a vacuum system for solvent drying, Protti et al. (2017) utilized a nitrogen stream (Table 4), like it was used in our DOFS extraction protocol. Therefore, our experiments suggest that the major loss for THC and CBD on DOFS have occurred by affinity with the filter paper. Although Mercolini et al. (2013) and Kyriakou et al. (2016) have reported > 80% recoveries for THC from DBS in Whatman 903® filter papers, these results were not reproducible using our DOFS extraction protocol. Our findings corroborate Stoykova et al. (2016) previous results for THC extraction using DOFS and Thomas et al. (2012) findings for THC extraction from DBS.

These limitations for THC and CBD recoveries from the filter paper have impacted on the LOD definition for our method. The LC–MS/MS instrument would be capable of detecting THC concentrations as low as 0.5 ng/mL, experimentally tested. However, to achieve this concentration after DOFS extraction with 25% recovery, a minimum THC concentration of 2 ng/mL in oral fluid (the established LOD for THC) is necessary. For CBD, the LOD was established at 4 ng/mL. Moreover, the recovery issues have impaired a quantitative analysis method validation. SAMHSA (SAMHSA 2019) have published a Mandatory Guideline for Federal Workplace Drug Testing Programs, for oral fluid samples, recommending a minimum of 80% recovery for THC from oral fluid collection devices, for confirmatory methods. Using this guideline as a parameter, the THC recovery percent from DOFS (25%) does not fit this requirement.

However, besides these limitations, the DOFS sampling method still provide advantages as a qualitative method for CBD and for THC identification and screening. For instance, drugs of abuse screening methods – using liquid chromatography and mass spectrometry—have been proposed for dried spots sampling using blood (Ambach et al. 2014; Chepyala et al. 2017) and urine (Michely et al. 2017; Pablo et al. 2020) as biological matrices. Studies in blood did not included cannabinoids in their panel of drugs, and, in urine, the metabolite 11-nor-9-Carboxy-Δ9-THC (THC-COOH) has been included with a LOD of 50 ng/mL (Pablo et al., 2020). Therefore, oral fluid presents advantages in demonstrating recent drug abuse.

Our DOFS qualitative method has demonstrated a low LOD for THC (2 ng/mL), which fits SAMHSA cut-off criteria recommendation for screening tests (4 ng/mL) in oral fluid (SAMHSA 2019). In addition, the qualitative confirmation analysis for THC and CBD on DOFS can occur concomitantly with the quantitative analysis for other drugs of abuse (Gorziza et al. 2020), in a single extraction method and detection instrument. Thus, when analyzing immunochromatographic screening tests, often used for roadside drug detection, there is a great variability, particularly for THC, concerning its cut-offs values, sensibility, specificity and accuracy, between different devices brands (Dobri et al. 2019). Therefore, insufficient oral fluid volume, device usability (e.g. testing time, failed tests and test reading) and instability due to cold weather have been reported as limitations of immunochromatographic screening tests for THC (Dobri et al. 2019). Comparatively, DOFS qualitative method provides THC and CBD precise identification with adequate sensitivity, fitting guidelines recommendations for LOD. In addition, considering its demonstrable stability (70% in 35 days, for both THC and CBD compounds), DOFS sampling is a suitable alternative for situations that require long distances sample collection and transportation, such as the roadside screening for suspected drivers under drug influence. Thus, DOFS is a low-cost procedure, requiring only a common polypropylene tube, a pipette tip and the filter paper, and it can be an alternative for a large demand of samples. The liquid–liquid extraction for DOFS also presents a lower cost than using a SPE cartridge, therefore saving time and reagents (solvents, nitrogen gas and chemical waste) in the extraction protocol for oral fluid.

To overcome the recovery limitation that we have observed in DOFS extraction for THC and for CBD, we have studied a new extraction method with WAX-S tips, as a suggestion for an alternative quantitative analysis. The WAX-S tips method utilizes liquid oral fluid, but it requires a low sample volume (50 µL), like DOFS. This advantage would allow for concomitant analysis between DOFS and WAX-S tips: the use of DOFS for THC and CBD sensitive screening, followed by WAX-S tips quantitative analysis if necessary.

WAX-S tips are disposable cartridges, similar to SPE techniques, but coupled to a micropipette. These tips contain a packed solid-phase sorbent in which the mixture of oral fluid and acetonitrile interact with during sample extraction through liquid aspiration and dispensing. Like DOFS, this method requires small amount of solvents (100 µL). In addition, the extraction procedure demands only a short period of time and dispenses the need of solvent evaporation, therefore reducing costs. The WAX-S tips method improved drug recoveries in oral fluid (Gorziza et al. 2020). In our study with THC and CBD detection in oral fluid, WAX-S tips also increased the drug recovery average, from 25% in DOFS to a 90%, with an average of 82% of process efficiency (Table 3). However, it must be noted that this study was performed with freshly spiked oral fluid, immediately extracted. In practical routine testing such as DUIC cases, oral fluid will be collected and transported to the laboratory for analysis. Oral fluid collection can be proceeded by direct spitting into a tube (like it was conducted in this study) or using a collection device constituted of a swab pad that conducts the oral fluid into a tube containing buffer and preservatives. While a collection device simplifies the procedure while helping with oral fluid viscosity and drug recoveries in general, increased absorptivity to collection devices is observed for lipophilic drugs, like THC (Crouch 2005). For instance, Langel et al. (2008) evaluated 9 different devices for oral fluid collection and drug recoveries, and substantial differences were found between the devices, especially for THC. In conclusion, WAX-S tips extraction is a fast and promising extraction method for cannabinoids in oral fluid should be further studied for a quantitative analysis combined with adequate oral fluid collection, following the identification of THC and CBD in DOFS.

## Conclusions

Oral fluid is a valuable matrix to detect cannabis recent use, effective in situations such as the screening of DUIC cases. Although THC and CBD compounds have been extensively studied in oral fluid, this is the first time that these compounds were studied for an analytical method validation in DOFS to ensure its applicability. Dried spots are a low-cost sampling method, which has demonstrated improved stability when compared to liquid samples. This advantage facilitates on-site collection and sample transportation, particularly required for long distances collections such as DUIC cases. A complete qualitative method validation is presented for THC and CBD confirmation in DOFS. As recovery issues have impaired a quantitative method validation in this study, which corroborates previous findings for THC in dried matrices, an alternative extraction method (WAX-S tips) is demonstrated to improve THC and CBD recoveries, and therefore it is suggested for further complementary quantitative studies.

## Data Availability

The data analyzed in this study are available from the corresponding author on reasonable request.

## Abbreviations

THC: Δ9-Tetrahydrocannabinol
CBD: Cannabidiol
DMS: Dried matrix spots
DOFS: Dried oral fluid spots
DBS: Dried blood spots
WAX-S: Weak Anion Exchange and Salt
DUIC: Driving under the influence of cannabis
DRUID: Driving under the influence of drugs
SAMHSA: Substance Abuse and Mental Health Services Administration
